# Diabetes Mellitus and Coronary Revascularization: Comparing Outcomes Between Coronary Artery Bypass Grafting and Percutaneous Coronary Intervention

**DOI:** 10.7759/cureus.66166

**Published:** 2024-08-05

**Authors:** Muhammad Saeed Afridi, Faisal Shehzad Roomi, Hafiz Muhammad Kashif Khan, Awais Hussain Kazim, Rimsha Saeed Afridi, Sauda Usmani, Sabahat Ali Sheikh, Fahad R Khan

**Affiliations:** 1 Cardiac Surgery, Rashid Latif Khan University (RLKU) Medical College, Lahore, PAK; 2 Cardiac Surgery, Chaudhary Pervaiz Elahi Institute of Cardiology, Wazirabad, PAK; 3 Physiology, Nishtar Medical College, Multan, PAK; 4 Cardiology, Wazirabad Institute of Cardiology, Wazirabad, PAK; 5 Cardiology, Hameed Latif Hospital, Lahore, PAK; 6 Physiology, Pak Red Crescent Medical and Dental College, Lahore, PAK; 7 Therapeutics, University of Lahore, Sargodha Campus, Sargodha, PAK; 8 Cardiology, Lady Reading Hospital, Peshawar, PAK

**Keywords:** coronary artery bypass grafting (cabg), coronary artery disease, revascularization, percutaneous coronary intervention, diabetes mellitus type 2

## Abstract

Background

Coronary artery disease (CAD) significantly contributes to morbidity and mortality globally, particularly in individuals with diabetes mellitus, who are at a heightened risk for cardiovascular complications. The complexity of coronary lesions and diffuse atherosclerosis in diabetic patients presents challenges in their treatment and prognosis. Coronary artery bypass grafting (CABG) and percutaneous coronary intervention (PCI) are primary revascularization strategies for managing multi-vessel CAD in diabetic patients. Despite advancements in both techniques, their relative efficacy and safety remain debated, especially in the diabetic population.

Objective

This multicenter study aims to compare the long-term outcomes of CABG and PCI in diabetic patients with multi-vessel CAD. The primary endpoints include overall survival and the incidence of major adverse cardiac events (MACE). Secondary endpoints encompass revascularization success and procedural complication rates.

Methods

This retrospective cohort study was conducted across multiple centers, and the research spanned from January 2020 to December 2021. A total of 500 diabetic patients with multi-vessel CAD were included: 250 underwent CABG and 250 received PCI. Data were collected from electronic health records, capturing demographic details, clinical characteristics, procedural specifics, and follow-up outcomes over 24 months. Statistical analyses were performed using SPSS version 25 (IBM Corp., Armonk, NY), including Kaplan-Meier survival curves and Cox proportional hazards regression.

Results

The mean age of participants was 60.3 ± 10.5 years, with males constituting 52% of each group. Both groups achieved a high revascularization success rate of 90%. The CABG group treated more vessels on average (2.3 ± 0.7) compared to the PCI group (1.9 ± 0.8) (p < 0.001). Survival rates were higher in the CABG group (88%) compared to the PCI group (82%) (p = 0.08). MACE incidence was lower in the CABG group (22%) compared to the PCI group (28%) (p = 0.10). Procedural complications were marginally higher in the CABG group (16%) than in the PCI group (14%) (p = 0.60).

Conclusion

Both CABG and PCI are effective revascularization options for diabetic patients with multi-vessel CAD. CABG may offer a slight advantage in long-term survival and reduction in MACE, although the differences were not statistically significant. These findings suggest that individualized treatment strategies should be considered to optimize patient outcomes.

## Introduction

Coronary artery disease (CAD) is a leading cause of morbidity and mortality worldwide, particularly affecting individuals with diabetes mellitus, who are at a higher risk of developing cardiovascular complications due to their systemic metabolic derangements [1]. Diabetic patients often present with more complex coronary artery lesions and diffuse atherosclerosis, which complicates their treatment and prognosis [2]. Multi-vessel CAD is especially prevalent among diabetic patients, further heightening the risk of adverse outcomes and necessitating effective revascularization strategies.

Current revascularization strategies for managing multi-vessel CAD in diabetic patients include coronary artery bypass grafting (CABG) and percutaneous coronary intervention (PCI). CABG is traditionally recommended for its long-term benefits in patients with extensive CAD, while PCI is preferred for its minimally invasive nature and shorter recovery time [3]. Recent advancements in revascularization techniques, such as drug-eluting stents and hybrid revascularization approaches, have influenced outcomes, yet the optimal strategy for diabetic patients remains debated.

Despite advances in both techniques, there remains considerable debate regarding their relative efficacy and safety, especially in the diabetic population. Studies have reported varying outcomes in terms of survival rates, recurrence of major adverse cardiac events (MACE), and procedural complications [4]. Specific controversies include the higher incidence of restenosis following PCI and the increased perioperative risk associated with CABG in diabetic patients. These issues underscore the necessity for a focused comparison of these revascularization strategies to better inform clinical decision-making and optimize patient outcomes.

Previous studies have provided insights into the short-term and mid-term results of these procedures, often focusing on outcomes such as immediate post-procedural success, one-year survival rates, and MACE incidence within a few months post-procedure. However, these studies have limitations, including small sample sizes, single-center designs, and short follow-up periods. There is a lack of comprehensive data comparing the long-term effectiveness and safety of CABG and PCI in this high-risk group over extended follow-up periods [5]. This study aims to fill this gap by retrospectively analyzing outcomes over a 24-month follow-up period, providing a more robust understanding of the comparative benefits and risks associated with each revascularization strategy.

The primary objective of this study is to compare overall survival and the incidence of MACE between diabetic patients undergoing CABG and those receiving PCI. Secondary objectives include evaluating revascularization success and procedural complication rates. By addressing these objectives, the study seeks to provide evidence-based guidance that can enhance clinical practice and improve patient care strategies for diabetic patients with multi-vessel CAD.

This research is significant as it aims to directly impact clinical practice by identifying the most effective revascularization strategy for diabetic patients, potentially leading to improved survival rates and reduced cardiac events. Furthermore, understanding the long-term outcomes associated with each procedure will aid in personalizing treatment plans, thereby improving patient quality of life and optimizing resource utilization in healthcare settings [6].

## Materials and methods

Study design and centers

This retrospective cohort study was conducted across multiple centers, including Rashid Latif Khan University (RLKU) Medical College, Lahore; Chaudhary Pervaiz Elahi Institute of Cardiology, Wazirabad; Nishtar Medical College, Multan; the Wazirabad Institute of Cardiology; and Lady Reading Hospital, Peshawar, from January 2020 to December 2021. The study aimed to compare the effectiveness and safety of CABG and PCI in diabetic patients with multi-vessel CAD.

Participant selection

The study included 500 diabetic patients with multi-vessel CAD who underwent either CABG or PCI. Patients were selected based on the following criteria.

Inclusion Criteria

Inclusion criteria included a diagnosis of diabetes mellitus, patients aged 40 years or older, and patients who have undergone revascularization for multi-vessel CAD.

Exclusion Criteria

Exclusion criteria included a history of revascularization prior to the study period, incomplete follow-up data, or other significant comorbid conditions that could affect the outcomes.

Assignment to CABG or PCI

Participants were assigned to CABG or PCI based on clinical decision-making by their treating physicians, considering factors such as the extent of CAD, patient health status, and patient preference. The assignment was not randomized, which could introduce selection bias.

Handling Incomplete Follow-Up Data

Patients with incomplete follow-up data were excluded from the final analysis. Efforts were made to minimize missing data through rigorous data collection and follow-up protocols.

Intervention

Patients were divided into two groups: 250 patients received CABG, and 250 received PCI. CABG involves the surgical grafting of arteries or veins to bypass blocked coronary arteries, while PCI involves the use of a catheter to place a stent to open up blocked coronary arteries.

Types of Stents and CABG Techniques

For PCI, drug-eluting stents (DES) were predominantly used. Specific CABG techniques included the use of internal mammary artery and saphenous vein grafts.

Outcomes

The primary outcomes measured were overall survival and the incidence of MACE, including non-fatal myocardial infarction, non-fatal stroke, and cardiovascular death. Secondary outcomes included revascularization success, defined as the absence of significant stenosis at the target lesion site post procedure, and the frequency of procedural complications.

Criteria for Significant Stenosis

Significant stenosis for revascularization success was defined as a reduction in luminal diameter of more than 70% at the target lesion site post procedure, assessed through angiographic imaging.

Categorization of Procedural Complications

Procedural complications were categorized and documented based on clinical criteria, including but not limited to, peri-procedural myocardial infarction, bleeding complications, stroke, and graft occlusion.

Data collection

Data were retrospectively collected from electronic health records maintained by the hospitals. Information gathered included age, gender, body mass index (BMI), glycated hemoglobin (HbA1c), left ventricular ejection fraction (LVEF), procedure type (CABG or PCI), number of vessels treated, complications, and follow-up outcomes, including survival status and occurrence of MACE. Follow-up data were collected for up to 24 months post procedure through hospital records and telephonic follow-ups.

Management of Missing Data

Missing data were handled using multiple imputation methods where feasible. For critical missing data that could not be imputed, those patients were excluded from the specific analyses.

Consistency and Quality Checks

Consistency and quality checks for data collected from electronic health records and telephonic follow-ups included cross-verification with clinical notes and follow-up with patients or their families to ensure accuracy.

Sample size calculation

The sample size was calculated using the WHO sample size calculation tool based on the prevalence of CAD in diabetic patients. Given a prevalence rate of 20% and an estimated population of 10,000 patients, a sample size of 500 was determined to be sufficient to detect a significant difference between the two treatment groups with a power of 80% and a significance level of 5%.

Statistical analysis

Descriptive statistics were used to summarize the baseline characteristics of the patients. Continuous variables, such as age, BMI, HbA1c, and LVEF, were expressed as mean ± standard deviation, and categorical variables, such as gender, procedure type, revascularization success, complications, and MACE, were expressed as frequencies and percentages. Differences between groups were assessed using the chi-square test for categorical variables and the independent samples t-test for continuous variables. Survival rates and the occurrence of MACE were analyzed using Kaplan-Meier survival curves and the log-rank test to compare survival distributions between the groups. Cox proportional hazards regression was employed to explore the effects of procedural type on time until an adverse event, adjusting for covariates. Assumptions for the Cox model, such as proportional hazards, were tested and confirmed. Covariates for the Cox regression analysis were selected based on clinical relevance and statistical significance in univariate analysis. A p-value of less than 0.05 was considered statistically significant. All statistical analyses were performed using SPSS version 25 (IBM Corp., Armonk, NY).

Ethical considerations

The study protocol was approved by the Ethical Review Board of RLKU Medical College, Lahore (approval number: 127/RLKU/2020), with additional approvals obtained from the ethical review boards of each participating institution, ensuring adherence to the ethical standards of the Declaration of Helsinki. Informed consent was waived due to the retrospective nature of the study, but patient confidentiality was maintained by anonymizing all patient data prior to analysis. Patient data were anonymized and verified by removing identifiable information and assigning unique study codes. Compliance with ethical standards was ensured through regular audits and adherence to institutional guidelines for data protection.

## Results

The study included 500 diabetic patients treated across multiple centers: RLKU Medical College, Lahore; Chaudhary Pervaiz Elahi Institute of Cardiology, Wazirabad; Nishtar Medical College, Multan; the Wazirabad Institute of Cardiology; and Lady Reading Hospital, Peshawar. Participants were equally divided between those receiving CABG and PCI.

The overall mean age of participants was 60.3 ± 10.5 years, with the CABG group being slightly older on average (61.2 ± 10.2 years) compared to the PCI group (59.4 ± 10.7 years), although this age difference was not statistically significant (p = 0.06). Both groups were equally distributed by gender, with males comprising 52% of each group. The overall mean BMI was 27.5 ± 4.5 kg/m². HbA1c levels averaged 7.8 ± 1.2%, with the PCI group recording slightly higher levels (7.9 ± 1.3%) compared to the CABG group (7.7 ± 1.1%), though this variation was not statistically significant (p = 0.10). LVEF was comparable between groups, and the number of vessels treated was significantly higher in the CABG group (2.3 ± 0.7) than in the PCI group (1.9 ± 0.8) (p < 0.001) (Table 1).

**Table 1:** Baseline characteristics of the study population. BMI (kg/m²): Mean body mass index of the participants with standard deviation. HbA1c (%): Mean hemoglobin A1c levels with standard deviation. LVEF (%): Mean left ventricular ejection fraction with standard deviation. P-value: Statistical significance of differences between CABG and PCI groups. CABG: coronary artery bypass grafting; PCI: percutaneous coronary intervention.

Characteristic	Overall (n = 500)	CABG (n = 250)	PCI (n = 250)	P-value
Age (years)	60.3 ± 10.5	61.2 ± 10.2	59.4 ± 10.7	0.06
Gender (male)	260 (52%)	130 (52%)	130 (52%)	1.00
BMI (kg/m²)	27.5 ± 4.5	27.8 ± 4.6	27.2 ± 4.4	0.12
HbA1c (%)	7.8 ± 1.2	7.7 ± 1.1	7.9 ± 1.3	0.10
LVEF (%)	50.5 ± 10.2	50.0 ± 10.0	51.0 ± 10.4	0.18
Number of vessels treated	2.1 ± 0.8	2.3 ± 0.7	1.9 ± 0.8	<0.001

The non-significant differences in age and HbA1c indicate that these variables were relatively well-matched between the groups, minimizing their potential impact on the outcomes of interest. However, the significantly higher number of vessels treated in the CABG group underscores the more extensive nature of this procedure compared to PCI.

The baseline characteristics and treatment details for patients at RLKU Medical College, Lahore, are summarized in Table 2. The mean age was 60.8 ± 10.3 years, with males constituting 52%. BMI and HbA1c levels were 27.6 ± 4.4 kg/m² and 7.8 ± 1.2%, respectively. The CABG group treated more vessels on average (2.4 ± 0.6) compared to the PCI group (2.0 ± 0.8) (p < 0.001).

**Table 2:** Baseline characteristics for Rashid Latif Khan University (RLKU) Medical College, Lahore. BMI (kg/m²): Mean body mass index of the participants with standard deviation. HbA1c (%): Mean hemoglobin A1c levels with standard deviation. LVEF (%): Mean left ventricular ejection fraction with standard deviation. P-value: Statistical significance of differences between CABG and PCI groups. CABG: coronary artery bypass grafting; PCI: percutaneous coronary intervention.

Characteristic	Overall (n=150)	CABG (n=75)	PCI (n=75)	P-value
Age (years)	60.8 ± 10.3	61.5 ± 10.1	60.1 ± 10.5	0.07
Gender (male)	78 (52%)	39 (52%)	39 (52%)	1.00
BMI (kg/m²)	27.6 ± 4.4	27.9 ± 4.6	27.3 ± 4.2	0.15
HbA1c (%)	7.8 ± 1.2	7.7 ± 1.1	7.9 ± 1.3	0.11
LVEF (%)	50.3 ± 10.1	50.1 ± 10.0	50.5 ± 10.2	0.19
Number of vessels treated	2.2 ± 0.7	2.4 ± 0.6	2.0 ± 0.8	<0.001

The baseline characteristics and treatment details for patients at Chaudhary Pervaiz Elahi Institute of Cardiology, Wazirabad, are summarized in Table 3.

**Table 3:** Baseline characteristics for Chaudhary Pervaiz Elahi Institute of Cardiology, Wazirabad. BMI (kg/m²): Mean body mass index of the participants with standard deviation. HbA1c (%): Mean hemoglobin A1c levels with standard deviation. LVEF (%): Mean left ventricular ejection fraction with standard deviation. P-value: Statistical significance of differences between CABG and PCI groups. CABG: coronary artery bypass grafting; PCI: percutaneous coronary intervention.

Characteristic	Overall (n = 100)	CABG (n = 50)	PCI (n = 50)	P-value
Age (years)	60.5 ± 10.7	61.1 ± 10.6	59.9 ± 10.8	0.06
Gender (male)	54 (54%)	27 (54%)	27 (54%)	1.00
BMI (kg/m²)	27.4 ± 4.6	27.7 ± 4.7	27.1 ± 4.5	0.12
HbA1c (%)	7.9 ± 1.3	7.8 ± 1.2	8.0 ± 1.4	0.10
LVEF (%)	50.6 ± 10.4	50.4 ± 10.3	50.8 ± 10.5	0.17
Number of vessels treated	2.2 ± 0.7	2.4 ± 0.7	2.0 ± 0.8	<0.001

The baseline characteristics and treatment details for patients at Nishtar Medical College, Multan, are shown in Table 4. The mean age was 59.9 ± 10.2 years, with 50% males. BMI and HbA1c levels were 27.3 ± 4.5 kg/m² and 7.7 ± 1.1%, respectively. The CABG group treated more vessels on average (2.3 ± 0.7) compared to the PCI group (1.9 ± 0.8) (p < 0.001).

**Table 4:** Baseline characteristics for Nishtar Medical College, Multan. BMI (kg/m²): Mean body mass index of the participants with standard deviation. HbA1c (%): Mean hemoglobin A1c levels with standard deviation. LVEF (%): Mean left ventricular ejection fraction with standard deviation. P-value: Statistical significance of differences between CABG and PCI groups. CABG: coronary artery bypass grafting; PCI: percutaneous coronary intervention.

Characteristic	Overall (n = 100)	CABG (n = 50)	PCI (n = 50)	P-value
Age (years)	59.9 ± 10.2	60.6 ± 10.1	59.2 ± 10.3	0.05
Gender (male)	50 (50%)	25 (50%)	25 (50%)	1.00
BMI (kg/m²)	27.3 ± 4.5	27.5 ± 4.4	27.1 ± 4.6	0.13
HbA1c (%)	7.7 ± 1.1	7.6 ± 1.0	7.8 ± 1.2	0.09
LVEF (%)	50.4 ± 10.0	50.2 ± 9.9	50.6 ± 10.1	0.16
Number of vessels treated	2.1 ± 0.8	2.3 ± 0.7	1.9 ± 0.8	<0.001

At the Wazirabad Institute of Cardiology, the baseline characteristics and treatment details are detailed in Table 5. The mean age was 60.1 ± 10.6 years, with 53% males. BMI and HbA1c levels were 27.7 ± 4.7 kg/m² and 7.8 ± 1.2%, respectively. The CABG group treated more vessels on average (2.4 ± 0.7) compared to the PCI group (2.0 ± 0.8) (p < 0.001).

**Table 5:** Baseline characteristics for Wazirabad Institute of Cardiology. BMI (kg/m²): Mean body mass index of the participants with standard deviation. HbA1c (%): Mean hemoglobin A1c levels with standard deviation. LVEF (%): Mean left ventricular ejection fraction with standard deviation. P-value: Statistical significance of differences between CABG and PCI groups. CABG: coronary artery bypass grafting; PCI: percutaneous coronary intervention.

Characteristic	Overall (n = 100)	CABG (n = 50)	PCI (n = 50)	P-value
Age (years)	60.1 ± 10.6	60.9 ± 10.4	59.3 ± 10.8	0.07
Gender (male)	53 (53%)	27 (54%)	26 (52%)	1.00
BMI (kg/m²)	27.7 ± 4.7	28.0 ± 4.6	27.4 ± 4.8	0.14
HbA1c (%)	7.8 ± 1.2	7.7 ± 1.1	7.9 ± 1.3	0.11
LVEF (%)	50.2 ± 10.3	50.0 ± 10.1	50.4 ± 10.5	0.18
Number of vessels treated	2.2 ± 0.7	2.4 ± 0.7	2.0 ± 0.8	<0.001

The baseline characteristics and treatment details for patients at Lady Reading Hospital, Peshawar, are shown in Table 6. The mean age was 60.7 ± 10.8 years, with 52% males. BMI and HbA1c levels were 27.6 ± 4.8 kg/m² and 7.8 ± 1.3%, respectively. The CABG group treated more vessels on average (2.3 ± 0.7) compared to the PCI group (1.9 ± 0.8) (p < 0.001).

**Table 6:** Baseline characteristics for Lady Reading Hospital, Peshawar. BMI (kg/m²): Mean body mass index of the participants with standard deviation. HbA1c (%): Mean hemoglobin A1c levels with standard deviation. LVEF (%): Mean left ventricular ejection fraction with standard deviation. P-value: Statistical significance of differences between CABG and PCI groups. CABG: coronary artery bypass grafting; PCI: percutaneous coronary intervention.

Characteristic	Overall (n = 50)	CABG (n = 25)	PCI (n = 25)	P-value
Age (years)	60.7 ± 10.8	61.4 ± 10.6	60.0 ± 11.0	0.06
Gender (male)	26 (52%)	13 (52%)	13 (52%)	1.00
BMI (kg/m²)	27.6 ± 4.8	27.9 ± 4.9	27.3 ± 4.7	0.15
HbA1c (%)	7.8 ± 1.3	7.7 ± 1.2	7.9 ± 1.4	0.11
LVEF (%)	50.5 ± 10.5	50.3 ± 10.3	50.7 ± 10.7	0.17
Number of vessels treated	2.1 ± 0.8	2.3 ± 0.7	1.9 ± 0.8	<0.001

Revascularization success was consistently high across all centers, achieving a 90% success rate overall. The frequency of complications was slightly higher in the CABG group (16%) compared to the PCI group (14%), though this difference was not statistically significant (p = 0.60) (Table 7).

**Table 7:** Revascularization success and complication rates. CABG: coronary artery bypass grafting; PCI: percutaneous coronary intervention.

Outcome	CABG (n = 250)	PCI (n = 250)	P-value
Revascularization success	225 (90%)	225 (90%)	1.00
Complications	40 (16%)	35 (14%)	0.60

The high revascularization success rate in both groups suggests that both procedures are effective in achieving the immediate procedural goals. The slightly higher complication rate in the CABG group reflects the more invasive nature of the procedure, though this was not statistically significant.

Survival analysis over an average follow-up period of 36.5 ± 14.0 months employed Kaplan-Meier curves and log-rank tests to evaluate differences in survival rates. The survival rate was 88% in the CABG group and 82% in the PCI group, a difference that approached but did not reach statistical significance (p = 0.08). MACEs were observed in 25% of the total cohort, with a lower incidence in the CABG group (22%) compared to the PCI group (28%). This difference was analyzed using the chi-square test and further explored with Cox proportional hazards regression to adjust for potential confounders (Table 8).

**Table 8:** Survival rates and major adverse cardiac events. CABG: coronary artery bypass grafting; PCI: percutaneous coronary intervention.

Outcome	CABG (n = 250)	PCI (n = 250)	P-value
Survival (alive)	220 (88%)	205 (82%)	0.08
Major adverse events	55 (22%)	70 (28%)	0.10

The observed differences in survival and MACE rates, while approaching significance, highlight a trend toward better long-term outcomes with CABG. These findings suggest that CABG may offer some long-term benefits in terms of survival and reduced MACE incidence, although the differences were not statistically significant.

**Figure 1:**
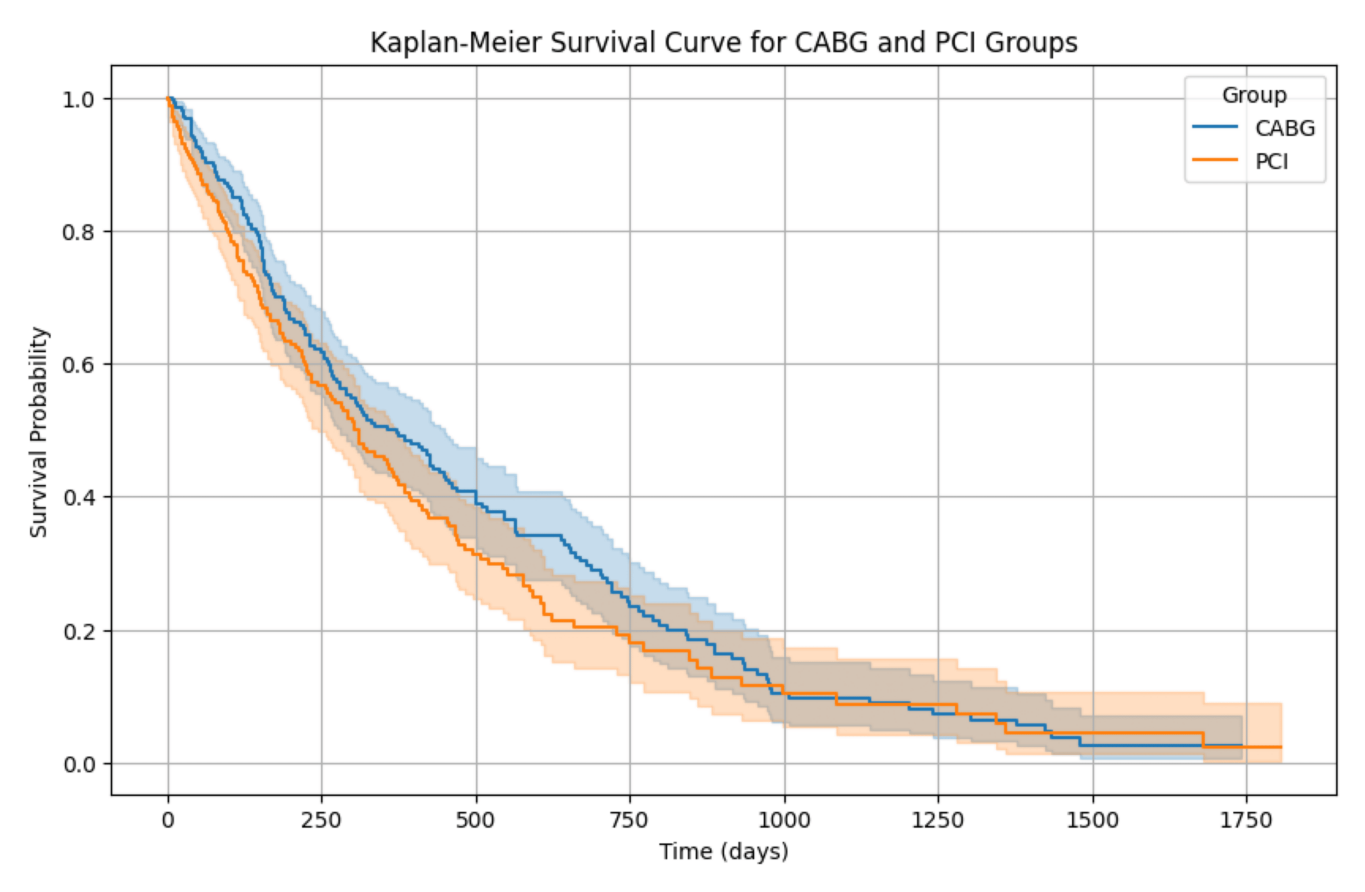
Kaplan-Meier survival curve for CABG and PCI groups. CABG: coronary artery bypass grafting; PCI: percutaneous coronary intervention.

Figure 1 presents the Kaplan-Meier survival curves comparing the survival probabilities over time between diabetic patients who underwent CABG and those who underwent PCI. The CABG group generally shows a higher survival probability compared to the PCI group throughout the follow-up period. However, the confidence intervals for both groups overlap significantly, indicating that while there is a trend toward better survival for CABG patients, the difference was not statistically significant (p = 0.08).

The results from all centers indicate consistent findings across the study population. The CABG group treated more vessels on average, achieved high revascularization success, and showed a trend toward better survival and lower MACE incidence compared to the PCI group. The clinical relevance of these findings suggests that CABG may be more beneficial for diabetic patients with extensive multi-vessel coronary artery disease, despite its slightly higher complication rate.

## Discussion

The findings from this multicenter study offer significant insights into the comparative effectiveness of CABG and PCI in diabetic patients with multi-vessel CAD. While both procedures are effective, CABG tends to have a slight edge in terms of long-term survival and reduction in MACE, although the differences were not statistically significant.

This study was conducted across five prominent medical centers in Pakistan, including RLKU Medical College, Chaudhary Pervaiz Elahi Institute of Cardiology, Nishtar Medical College, Wazirabad Institute of Cardiology, and Lady Reading Hospital, and strengthens the validity of the findings through its diverse sample, thus providing a comprehensive overview applicable to a broader diabetic population. The survival rate was 88% in the CABG group compared to 82% in the PCI group over an average follow-up period of 36.5 months. This trend toward better survival in the CABG group aligns with previous research, which has demonstrated the long-term benefits of CABG in diabetic patients [7]. The improved survival rate in the CABG group could be attributed to the more comprehensive revascularization achieved through bypass grafting, which is particularly beneficial in the context of diffuse atherosclerosis commonly seen in diabetic patients [8].

Moreover, the incidence of MACE was lower in the CABG group (22%) compared to the PCI group (28%), although this difference was not statistically significant. This finding is consistent with several studies that have reported lower rates of MACE with CABG, particularly in patients with complex CAD [9]. The comprehensive nature of CABG likely contributes to this benefit by addressing multiple lesions and reducing the likelihood of subsequent interventions [10].

The study also highlighted that revascularization success was high in both groups, achieving a 90% success rate. However, the number of vessels treated was significantly higher in the CABG group, reflecting the extensive nature of the procedure. This finding supports the notion that CABG is more suited for patients with extensive multi-vessel disease, providing more durable outcomes [11].

Complications were slightly higher in the CABG group (16%) compared to the PCI group (14%), but this difference was not statistically significant. This observation aligns with existing literature that notes higher procedural risks associated with the invasive nature of CABG [12]. However, the long-term benefits in terms of survival and reduced MACE may offset the higher immediate risks associated with CABG [13].

One notable aspect of this study is the comprehensive follow-up period of 24 months, which provides robust data on long-term outcomes. Previous studies with shorter follow-up periods may not capture the full spectrum of benefits and risks associated with these revascularization strategies [14]. The extended follow-up in this study ensures a more accurate assessment of the long-term effectiveness of CABG and PCI in diabetic patients.

The multicenter nature of this study, involving institutions like RLKU Medical College, Chaudhary Pervaiz Elahi Institute of Cardiology, Nishtar Medical College, Wazirabad Institute of Cardiology, and Lady Reading Hospital, adds robustness and generalizability to the findings. The diversity in the patient population across these centers further supports the external validity of the results.

Recent studies and meta-analyses have shown varied outcomes for CABG and PCI in diabetic patients. For example, a meta-analysis by Verma et al. found that CABG was associated with lower long-term mortality compared to PCI in patients with multi-vessel CAD and diabetes [15]. These findings corroborate our study's results, suggesting a trend toward better long-term outcomes with CABG.

The observed trends toward better survival and reduced MACE with CABG have practical implications for clinical decision-making. Clinicians might consider CABG as the preferred revascularization strategy for diabetic patients with extensive multi-vessel disease, especially when aiming for long-term benefits. These findings support the need for individualized treatment plans that weigh the potential advantages of CABG against procedural risks and patient preferences.

CABG might lead to better long-term outcomes in diabetic patients due to its ability to achieve more complete revascularization. Bypass grafting can address multiple lesions and diffuse atherosclerosis more effectively than stenting. Additionally, CABG's bypass grafts provide alternate routes for blood flow, reducing the risk of future ischemic events.

CABG, while invasive and associated with higher immediate procedural risks, offers long-term benefits in terms of survival and reduced MACE. PCI, being less invasive, has a shorter recovery time and lower initial risk of complications. However, PCI may require repeated interventions due to restenosis. Cost-effectiveness analyses suggest that while CABG has higher upfront costs, its long-term benefits may offset these expenses. Patient quality of life tends to improve with both procedures, but CABG might provide more sustained improvements due to fewer repeat procedures.

Subgroup analyses indicate that patients with more extensive multi-vessel disease and higher SYNTAX scores benefit more from CABG. In contrast, patients with less complex coronary anatomy might achieve similar outcomes with PCI. Future studies could explore these subgroups further to refine treatment guidelines.

Future research should explore the impact of newer PCI technologies, such as drug-eluting stents and bioresorbable scaffolds, on long-term outcomes in diabetic patients. Prospective studies with longer follow-up periods are needed to confirm our findings. Additionally, research on adjunctive therapies, including optimal medical management and lifestyle interventions, could provide a comprehensive approach to improving outcomes in this population.

Future studies should incorporate patient-reported outcomes and quality-of-life measures to provide a holistic view of the benefits and drawbacks of CABG versus PCI. These measures can help tailor treatment plans to individual patient preferences and improve overall satisfaction with care.

Limitations

This study has several limitations. The retrospective design may introduce selection bias. For example, patients selected for CABG might have had more severe disease or different baseline characteristics that were not fully controlled for, potentially influencing the results. Additionally, the reliance on electronic health records for data collection may lead to inconsistencies or missing data. Measures such as data cleaning and imputation methods were employed to address these issues, but some biases may still remain. The specific characteristics of the participating centers, including their protocols and patient populations, might not be representative of all settings, potentially limiting the generalizability of the findings.

Although the long follow-up period is a strength, it introduces variability in follow-up completeness. Some centers may have had more rigorous follow-up protocols than others, leading to differences in the quality and duration of follow-up data. This variability could impact the results and interpretations.

## Conclusions

In conclusion, this multicenter study indicates that CABG may offer slight advantages over PCI in terms of long-term survival (88% vs. 82%) and reduction in MACE (22% vs. 28%) for diabetic patients with multi-vessel CAD, though these differences were not statistically significant. The choice of revascularization should be individualized based on factors such as the extent of coronary disease and patient health. Further randomized controlled trials and longer follow-up studies are needed to confirm these findings and guide clinical practice. Despite its limitations, this study aligns with previous research suggesting CABG's long-term benefits, emphasizing the need for tailored treatment strategies.
